# Is CK7 a Prognostic Marker in Pulmonary LCNEC? Evidence from a Limited Cohort Study †

**DOI:** 10.3390/jpm15020067

**Published:** 2025-02-12

**Authors:** Hruy Menghesha, Donatas Zalepugas, Amina Camo, Georg Schlachtenberger, Konstantinos Grapatsas, Andres Amorin Estremadoyro, Fabian Doerr, Matthias Heldwein, Alexander Quaas, Servet Bölükbas, Gerardus Bennink, Joachim Schmidt, Khosro Hekmat

**Affiliations:** 1 Division of Thoracic Surgery, Department of General, Thoracic and Vascular Surgery, Bonn University Hospital, 53127 Bonn, Germany; 2 Department of Thoracic Surgery, Helios Clinic Bonn/Rhein-Sieg, 53123 Bonn, Germany; 3 Faculty of Medicine, University of Cologne, Joseph-Stelzmann-Strasse 20, 50931 Köln, Germany; 4 Department of General, Visceral and Thoracic Surgery, University Hospital of Cologne, 50937 Cologne, Germany; georg.schlachtenberger@uk-koeln.de (G.S.);; 5 Department of Thoracic Surgery, University Medical Center Essen-Ruhrlandclinic, Tüschener Weg 40, 45239 Essen, Germany; 6 Institute of Pathology, University Hospital of Cologne, 50937 Cologne, Germany; 7 Department of Cardiothoracic Surgery, Heart Center, University Hospital Cologne, Kerpener Strasse 62, 50937 Cologne, Germany

**Keywords:** immunohistochemistry, LCNEC, adenocarcinoma, lung cancer

## Abstract

**Objectives:** While the treatment of non-small-cell lung carcinoma has improved rapidly, the treatment of pulmonary large-cell neuroendocrine carcinoma (LCNEC) remains underdeveloped. The use of immunohistochemistry allows for accurate risk stratification. With our study, we investigated the outcome of patients with pulmonary LCNEC and analyzed whether CK7 correlates with long-term survival. **Methods:** We retrospectively collected the monocentric data of patients which underwent anatomical resection for lung cancer between January 2012 and December 2020. Patients that did not show pulmonary LCNEC or adenocarcinoma, had a positive resection margin, or underwent neoadjuvant therapy were excluded. The long-term survival rate of the LCNEC and adenocarcinoma groups were compared before and after propensity score matching. Furthermore, we performed survival analyses for a subgroup of LCNEC distinguished by CK7 expression, followed by Cox regression analyses. **Results:** A total of 466 patients were integrated for further analysis. The mean age was 65.3 ± 9.6 years. There were no significant differences between both groups regarding age, gender, or comorbidities. In terms of the UICC stage, the groups were equally distributed. Mean survival in the LCNEC group was significantly worse than in the adenocarcinoma group (LCENC: 36.4 ± 7.5 months; adenocarcinoma: 80.7 ± 8.1 months; *p*-value = 0.001). The mean survival rate was 19.23 ± 4.8 months in the CK7 expression group and 57.01 ± 8.5 months in the group without expression, which reached statistical significance (*p*-value = 0.019). **Conclusions:** Our study suggests that pulmonary LCNEC has a significantly worse prognosis than pulmonary adenocarcinoma. CK7 expression seems to be correlated with a worse outcome for the long-term survival rate of patients suffering from highly malignant pulmonary LCNEC.

## 1. Introduction

The term “lung cancer” represents multiple different subtypes of primary pulmonary malignoma. When combined, they are responsible for approximately 20% of all cancer-related deaths worldwide [1]. While the treatment of non-small-cell lung carcinoma (NSCLC) has improved rapidly over the past decade with the introduction of many different targeted therapeutic approaches, there is a group of neuroendocrine pulmonary neoplasms (NENs) for which the state of knowledge still appears limited [2]. The inhomogeneous group of pulmonary neuroendocrine tumors can be divided into low- and high-malignancy tumor entities [3]. While low-malignancy neuroendocrine lung tumors, represented by typical (TCs) and atypical carcinoids (ACs), regularly require a simple follow-up and no adjuvant therapy after resection in the operable stages [4], adjuvant systemic therapy is recommended for large-cell neuroendocrine carcinoma (LCNEC) even in the early stages [5]. Due to the lack of a definitive histopathologic definition of LCNEC and its prevalence of three percent of all lung cancer entities [6], scientific progress regarding this rare, highly malignant entity and the associated advancement of therapy has been slow. In 1985, LCNEC was recognized as a distinct clinicopathological entity by Hammond and Sause [7]. While the World Health Organization (WHO) classifies LCNEC as a neuroendocrine carcinoma [8], the clinical presentation and therapeutic management are often considered compatible with those of small-cell lung cancer (SCLC) [9]. However, LCNEC differs from SCLC in approximately 25% initial early-stage diagnoses and presents with more peripherally located primary lesions [10]. On the other hand, the National Comprehensive Cancer Network (NCCN) guidelines describe LCENC as a subtype of NSCLC and, accordingly, recommend stage-dependent therapy [11]. Nevertheless, the results of retrospective investigations show poor long-term survival despite radical surgery in early-stage LCNEC, as well as compared to the other subgroups of NSCLC—squamous cell carcinoma (SCC) and adenocarcinoma (ADC) [12,13].

Advanced analytical methods such as immunohistochemistry can be used for better specification. Immunohistochemistry utilizes the specific binding between an antibody and an antigen in order to detect specific antigens, most likely using a light microscope [14]. Immunohistochemistry is often used to differentiate the specific tumor entity or determine a metastatic tumor’s site of origin. In addition, it is providing increasing support in estimating the prognosis of specific disease progression, depending on the ability to visualize certain antigens, such as in testing for HER2 amplification in breast cancer [15].

Cytokeratins (CKs) are structural proteins of the cytoskeleton that can be visualized in the epithelia and tumor cells derived from the epithelia [15]. CKs are a family of intermediate filament proteins primarily expressed in epithelial cells, playing a critical role in maintaining cellular structure and integrity. These proteins are involved in various cellular processes, including mechanical support, signal transduction, and cellular differentiation. They are highly tissue-specific, with different CKs serving as markers to identify epithelial cell types and their pathological states. For example, CK8 and CK18 are commonly found in simple epithelia, while CK5 and CK14 are typically associated with stratified epithelia. Cytokeratins are useful as biomarkers in clinical diagnostics, particularly in identifying epithelial tumors, where their expression patterns can provide insights into the origin [16]. CK expression is usually retained in degenerated tumor cells. This allows for conclusions to be drawn about the origin of the tumor cells. This mechanism is regularly used in the diagnosis of metastases [17].

CK7 is widely used as a biomarker in the diagnosis of lung cancer, particularly in distinguishing different histologic subtypes and determining the primary site of metastatic tumors. CK7 is typically expressed in the epithelial cells of various organs, including the lungs, and its expression pattern can help identify the origin of tumors in the lungs. In non-small-cell lung cancer (NSCLC), CK7 is frequently expressed in ADC, while it tends to have negative or low expression in SCC [17]. Additionally, CK7 can be useful in differentiating primary lung cancers from metastases, as certain metastatic carcinomas such as those from the breast or colon may exhibit different CK7 expression patterns [18].

CK7 plays a significant role in the diagnostic differentiation of large-cell neuroendocrine carcinoma (LCNEC) of the lung. CK7 is often expressed in LCNEC, and its presence can help to distinguish this tumor from other small-cell lung carcinomas (SCLCs), which typically show negative or only weak expression of CK7 [19].

With the help of our monocentric retrospective study, we compare the long-term outcome of our patients suffering from LCNEC with that of patients suffering from the most common non-small-cell pulmonary malignancy. Secondly, we aim to investigate whether CK7, as one representative of the potential immunohistochemical markers, can be used as a prognostic factor for the long-term survival of LCNEC patients.

## 2. Patients and Methods

For our monocentric study, we collected the retrospective data of patients that underwent an anatomical resection with systematic lymphadenectomy after a diagnosis of lung cancer at one institution between January 2012 and December 2020. After the exclusion of patients that did not present with pulmonary LCNEC or pulmonary ADC, had a positive resection margin, or underwent neoadjuvant therapy, 466 patients were eligible for further analysis. These patients were distributed into the two groups according to their histological entity—LCNEC or ADC. Due to its retrospective character and the complete anonymization of patient data, the need for written informed consent and ethical approval was waived. We analyzed the tumor entity, common TNM classification, infiltration of resection margin (R0, R1, or R2), grading of malignancy (G1, G2 or G3), and vessel invasion (V1 or L1). Incomplete pathological reports were not included in analysis.

### 2.1. Diagnostic and Treatment

The preoperative staging of patients diagnosed with lung cancer or with reasonable suspicion of lung cancer was performed according to the European and German Lung Cancer Guidelines [20,21]. After visualization of a pulmonary lesion in a high-resolution computed tomography (hr-CT), positron emission tomography (PET-CT) and magnetic resonance imaging of the brain (cMRI) were performed and examined by experts from the fields of radiology and nuclear medicine. Pulmonary function testing with the determination of the diffusing capacity of carbon monoxide (DLCO) and forced expiratory volume in one second (FEV1) was used to assess functional operability. Each test result was evaluated for plausibility by an expert in Pneumology, in correlation with the patient’s clinic, the radiological findings, and the patient’s cooperation during lung function testing. The diagnostic and therapeutic procedures were discussed and determined repeatedly during the course of the patient’s treatment at a multidisciplinary tumor conference (MDT) at least once before and once after the operation. If surgery was indicated, surgery was exclusively performed by experienced surgeons, and the resection specimens were analyzed by lung cancer experts from the Department of Pathology. Patients with NSCLC stage IA-IB were treated only surgically and received a subsequent tumor follow-up. Patients at stage II or higher received adjuvant chemotherapy and/or chemoradiation therapy, as was recommended by the guidelines. Upon an assessment of the tumor size or the invasion of thoracic structures, a neoadjuvant therapy concept was regularly discussed and applied if indicated.

### 2.2. Immunohistochemical Staining

Immunohistochemical staining (IHC) on whole tumor blocks was conducted to assess the expression of cytokeratin 7 (CK 7). The following antibody was used: CE-certified Dako mouse clone OV-TL12/30, dilution of 1:6000, enzyme pre-treatment, stained on the automatic staining system Leica BOND-MAX including the Leica Bond Polymer Refine Detection Kit (Leica Biosystems, Wetzlar, Germany). We used normal lung tissue as an on-slide positive control. Analysis of the specimens was performed by one of the pathologists (A.Q.). CK7 of any cytoplasmatic intensity was classified according to their percentage of the stained tumor cells (0% stained tumor cells = negative, >1% stained tumor cells = positive). The photograph was taken on a Leica DM4000 B LED microscope at a magnification of 200× using the microscope-connected HV F202 camera (Hitachi, Chiyoda, Tokyo) and the software Diskus version 4.80.9941.

### 2.3. Statistical Analysis and Propensity Score Matching

Data were analyzed using the Pearson’s χ^2^ for nominal data and Fisher’s exact test or Student’s *t*-test for metric data depending on normal distribution after propensity score matching. When analyzing ADC vs. LCNEC, Welch’s *t*-test for unequal variances was used for the metric data before propensity score matching, as the sample size was very unequal. Normal distribution was evaluated using the Shapiro–Wilk test. Continuous variables were expressed as mean ± standard deviation. The 3-year survival rate was calculated from the date of surgery to the date of death or last patient contact. The mean survival rate was assessed by the Kaplan–Meier method and compared by the log-rank test. Additionally, we performed a propensity score matching (PSM) analysis. Over the course of the propensity score matching, patients were matched according to age, gender, lymph node status, lymphatic vessel invasion, blood vessel invasion, tumor size, smoking habits, distant metastases, comorbidities (coronary heart disease (CHD), chronic obstructive pulmonary disease (COPD)), and performance status (Karnofsky Index). A *p*-value < 0.05 was accepted as statistically significant. In order to evaluate the independence of CK7 as a prognostic factor for the long-term survival of LCNEC patients, a hazard ratio was calculated using Cox regression analysis.

## 3. Results

Out of 747 patients, 466 were eligible for further analysis after applying the above-mentioned exclusion criteria. The study population was divided into two groups—ADC (*n* = 450) and LCNEC (*n* = 16).

### 3.1. Baseline Characteristics

The baseline characteristics for the whole study population are summarized in Table 1. The mean age of the patients diagnosed with ADC was 65.2 ± 9.6 years, compared to 67.3 ± 10.9 years within the LCNEC group (*p* = 0.47). The gender distribution was equal between both groups, with a calculated *p*-value of 1.0. Both groups showed no significant differences in preoperative performance status measured by the Karnofsky Index (*p* = 0.572). The prevalence of coronary heart disease was higher in the ADC group (24%) than in the LCNEC group (12.5%), although the result did not reach statistical significance (*p* = 0.38). The situation was similar for arterial hypertension (aHT). In the ADC group, 55.6% of patients suffered from aHT, while in the LCNEC group, only 31.3% suffered from aHT (*p* = 0.073). Smokers were similarly represented in both groups, at approximately 60% (*p* = 1.0). Smoking habits, measured by pack-years, also showed no significant difference between the two groups (*p* = 0.683). The length of hospital stay was nearly the same in both groups, with 8.48 ± 4.7 days in ADC patients and 9.25 ± 5.1 days in LCNEC patients (*p* = 0.563). After the propensity score matching, the UICC stages, vessel invasion, and lymph nodal involvement were similarly distributed (Table 2), so we can assume that further survival analyses were not biased by any baseline differences.

### 3.2. Long-Term Survival Unmatched

**Figure 1:**
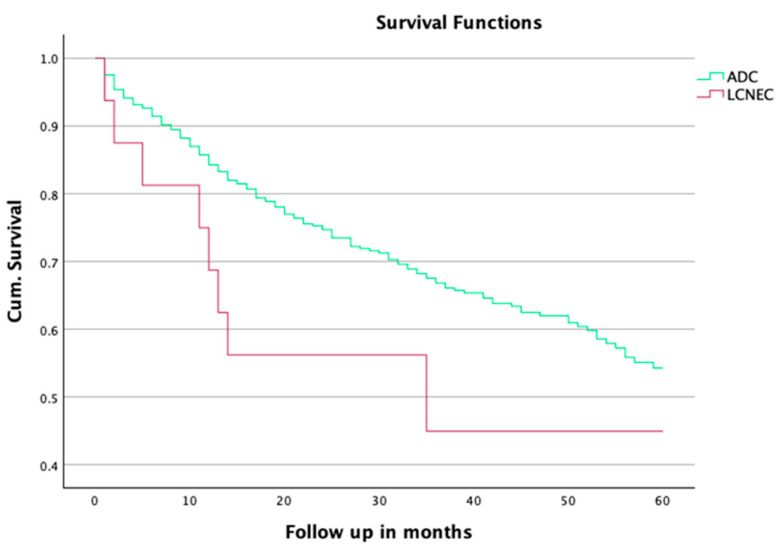
Overall survival before PSM.

The mean survival rate of the patients with ADC after an anatomical resection was 62.5 ± 2.3 months, significantly better compared to 36.4 ± 7.5 months in the LCNEC group (*p* = 0.011). The 3-year survival rates were significantly worse in the LCNEC group (27.3%) than in the ADC group (59.9%), with a *p*-value of 0.033. Mean and long-term overall survival rates before propensity score matching are presented in Table 3 and Figure 1.

### 3.3. Long-Term Survival After Propensity Score Matching Cohort

**Figure 2:**
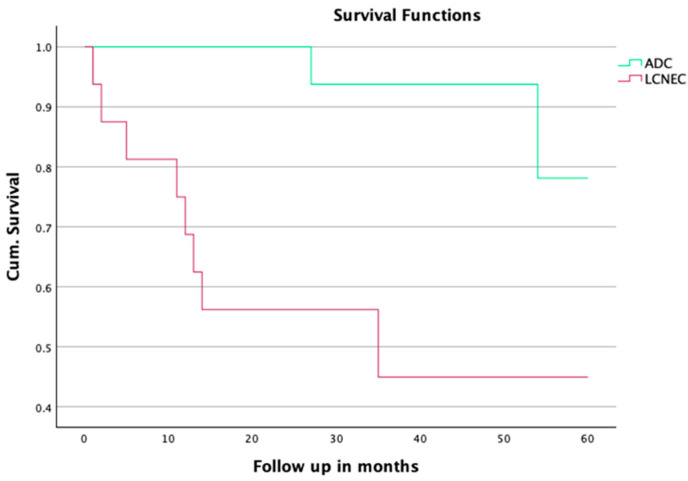
Overall survival after PSM.

The mean survival rate of the patients with ADC was 80.7 ± 8.1 months, compared to 36.4 ± 7.5 months in the LCNEC group (*p* = 0.001). The 3-year survival rate was 92.9% in the ADC group and 27.3% in the LCNEC group (*p* = 0.002). The mean and long-term overall survival rates for the propensity score matched cohort are presented in Table 3 and Figure 2.

### 3.4. Subgroup Analysis of LCNEC CK7+ vs. CK7−

The subgroup analysis was performed on the 16 patients who suffered from LCNEC. These were divided into the CK7-positive (CK7+) and CK7-negative (CK7−) groups, according to the CK7 status. There were 10 patients in the CK7+ group and 6 patients in the CK7− group. The baseline characteristics are summarized in Table 4. There were no significant differences between the groups of CK7+ and CK7− patients with regards to the UICC stage, blood vessel invasion, lymphatic vessel invasion, lymph node metastasis, or the expression of other immunohistochemical markers. Nevertheless, the group of CK7− patients showed a higher proportion of chromogranin A positivity (CK7+: 10.0%; CK7−: 66.6%), with a *p*-value of 0.08, indicating no statistically significant difference but suggesting a potential trend.

**Figure 3:**
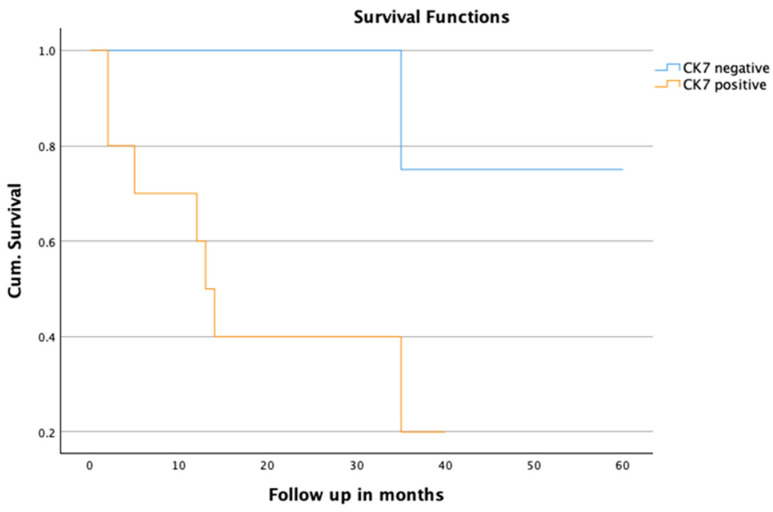
Subgroup analysis of LCNEC distinguished by CK7 status.

Subgroup analysis among the patients presenting with LCNEC showed that patients with CK7+ results in the immunohistochemical analysis had a significantly worse outcome in terms of mean survival rate (CK7+: 19.23 ± 4.8 months; CK7−: 57.01 ± 8.5 months, *p* = 0.019). Although the long-term survival rates suggest a clear trend, with a 3-year survival rate of 12.5 % in the CK7+ group and 75 % in the CK7− group (*p*-value = 0.067), the difference is not statistically significant. Cox regression analysis revealed a *p*-value of 0.014 and a hazard ratio of 9.02 for CK7 expression as a prognostic factor for OS in LCNEC patients. The data are presented in Table 5 and Figure 3 for further details.

## 4. Discussion

Pulmonary neuroendocrine neoplasms (NENs) represent a distinct and clinically significant subset of lung cancers, constituting approximately 20–30% of all NENs and accounting for about 25% of lung cancer cases [22]. Within this category, large-cell neuroendocrine carcinoma (LCNEC) emerges as a particularly aggressive subtype, representing roughly 3% of all lung cancer diagnoses. Historically grouped under non-small-cell lung cancer (NSCLC), LCNEC is now recognized as one of four neuroendocrine subtypes of pulmonary malignancies, reflecting its unique pathological and clinical characteristics [8].

Our investigation reveals pronounced differences in the mean survival outcomes between patients diagnosed with LCNEC and those with pulmonary adenocarcinoma (ADC). These disparities are evident even before propensity score matching and become significantly more pronounced post-matching, as demonstrated by highly significant *p*-values and a greater divergence in the Kaplan–Meier survival curves (Table 3, Figure 3). These findings corroborate the previous research, such as the work of Zou et al., who utilized data from the SEER database to demonstrate the inferior survival rates of LCNEC patients compared to other NSCLC subtypes. Zou et al. further highlighted the importance of adjuvant and neoadjuvant therapies in improving the outcomes for early-stage LCNEC patients, underscoring the necessity of aggressive and tailored treatment approaches [23].

### 4.1. The Role of Multidisciplinary Management

The data emphasize the critical role of a multidisciplinary team (MDT) in managing LCNEC, with particular attention to radical surgical interventions supported by adjuvant therapies, even at early stages. In our cohort, all the ADC patients received adjuvant therapy in accordance with the established guidelines when indicated. Among the LCNEC patients, four underwent adjuvant chemotherapy with platinum doublets over at least three cycles, and one patient additionally received radiotherapy with 60 Gy delivered via multi-field technique. Such comprehensive treatment regimens appear essential for mitigating the aggressive clinical behavior of LCNEC, which often parallels that of small-cell lung cancer (SCLC) in its rapid progression and poor prognosis.

### 4.2. LCNEC Classification and Therapeutic Strategies

The World Health Organization (WHO) classifies LCNEC as one of the four subtypes of pulmonary neuroendocrine neoplasms. While retaining its classification as a neuroendocrine variant of large-cell carcinoma, LCNEC’s aggressive nature and unique molecular profile differentiate it within the broader NSCLC category [24,25,26]. A pivotal Italian cohort study involving 144 patients reported poor survival rates in early-stage LCNEC, although outcomes were significantly improved with adjuvant chemotherapy, particularly at Stage I of the disease [12]. Similarly, Iyoda et al. demonstrated a 20–40% survival benefit for early-stage LCNEC patients treated with at least two cycles of platinum and etoposide [27].

Further supporting these observations, high-volume thoracic surgery centers have reported comparable survival benefits from chemotherapy in early-stage LCNEC cases [28]. Emerging molecular profiling studies have begun to unravel the genetic landscape of LCNEC, frequently identifying mutations in TP53 and RB1 as the key drivers of its aggressive phenotype. These insights underscore the necessity for multimodal treatment strategies that integrate systemic therapies with surgical intervention and pave the way for potential targeted therapeutic approaches.

### 4.3. Prognostic Value of CK7 Expression

Our subgroup analysis identified CK7 expression as a significant predictor of poor survival in LCNEC patients, with the CK7-positive individuals exhibiting markedly reduced mean survival compared to their CK7-negative counterparts (Figure 3). Notably, other immunohistochemical markers were evenly distributed within this subgroup, highlighting CK7 as a particularly salient prognostic marker. These findings align with studies by Leo et al., who demonstrated that the expression levels of CK7 and CK20 were associated with higher rates of lymph node metastasis, advanced tumor stage, and poorer outcomes in colorectal carcinoma [29]. Similarly, Hrudka et al. established CK7 as an independent prognostic indicator in colorectal cancer [30].

Immunohistochemical profiling is increasingly pivotal in the nuanced classification and prognostication of carcinomas, particularly within neuroendocrine neoplasms [31]. Despite its growing importance, such advanced analyses are not yet universally implemented across pathology centers. Centers of excellence should adopt these assessments as part of their diagnostic protocols to enhance tumor characterization and guide treatment planning.

Recent investigations into the role of CK7 have also explored its involvement in oncogenic processes, such as epithelial–mesenchymal transition (EMT) and chemoresistance. These studies suggest that CK7 may directly influence tumor progression and therapeutic responsiveness, further underscoring its potential as a biomarker for patient stratification and personalized treatment strategies.

### 4.4. Future Research Directions

The prognostic significance of CK7 expression in LCNEC warrants further exploration through multicenter studies involving larger patient cohorts. While our findings suggest a strong correlation, the limited sample size of our study precludes definitive conclusions. Validation of these results could establish CK7 as a valuable prognostic tool, aiding in clinical decision-making and enhancing patient counseling.

Additionally, molecular studies aimed at elucidating the mechanisms underlying CK7’s impact on tumor biology may reveal novel therapeutic targets. For instance, interventions designed to modulate CK7-associated pathways could offer promising avenues for improving the outcomes in LCNEC patients. Incorporating CK7 assessment into routine diagnostic workflows would facilitate more accurate risk stratification and enable the implementation of personalized therapeutic approaches.

Our findings underscore the aggressive clinical behavior of LCNEC and highlight the survival benefits of adjuvant chemotherapy, even in early-stage cases. The observed association between CK7 expression and poorer survival introduces a compelling avenue for future research, although larger and more diverse patient cohorts are necessary to confirm these findings. As a rare and highly malignant entity, LCNEC presents unique challenges that demand continued refinement in the diagnostic and therapeutic paradigms.

Future research should prioritize collaborative efforts to validate the prognostic value of CK7 and elucidate its molecular underpinnings. Advances in immunohistochemistry, precision oncology, and targeted therapy development will be instrumental in addressing the complexities of LCNEC and improving patient outcomes on a broader scale.

### 4.5. Limitations

This study is subject to several limitations that must be acknowledged. First, the small sample size of 16 patients significantly limits the generalizability of our findings. With such a small cohort, statistical power is reduced, and the ability to detect nuanced differences or draw robust conclusions is constrained. Second, selection bias may have influenced the results, as patients included in the study may not represent the broader population of individuals with LCNEC. This bias could result from the retrospective nature of the analysis and the criteria used to select participants. Third, the lack of external validation further restricts the applicability of our findings. Without corroboration from larger, independent cohorts or multicenter studies, the reliability and reproducibility of these results remain uncertain. Addressing these limitations in future research will be essential to confirm and expand upon the insights presented in this paper.

## 5. Conclusions

In conclusion, our findings suggest that LCNEC tends to result in a worse long-term survival rate compared to pulmonary adenocarcinoma, although international guidelines generally recommend adjuvant chemotherapy for LCNEC patients even at the early stages. Immunohistochemical analyses, while not solely used for precise differentiation from other conditions, could potentially aid in risk stratification. This could support improved decision-making within the multidisciplinary tumor boards and provide more informed guidance for patient care. While our study indicates that the CK7 status may influence outcomes in LCNEC, further investigation into other types of pulmonary carcinoma is warranted. A key limitation of our study is the small patient cohort, which may limit the broader applicability of our conclusions. However, we believe our findings could form a valuable basis for further research, particularly within the framework of national and international collaborations.

## Figures and Tables

**Table 1:** Baseline characteristics of ADC and LCNEC patients.

	Total (*n* = 466)	ADC (*n* = 450)	LCNEC (*n* = 16)	*p*-Value
Demographic data				
Age (years)	65.3 ± 9.6	65.2 ± 9.6	67.3 ± 10.9	0.47
Female (%)	47.2	47.1	50.0	1.0
BMI	25.9 ± 4.7	25.9 ± 4.7	24.8 ± 4.4	0.34
Preoperative fitness				
Karnofsky-Index	78.9 ± 12.5	78.9 ± 12.5	75.0 ± 7.1	0.572
Comorbidity				
COPD (%)	29.2	29.6	18.8	0.42
CHD (%)	23.6	24.0	12.5	0.38
Hypertension (%)	54.7	55.6	31.3	0.073
Smoking habit				
Smoking (%)	60.9	60.9	62.5	1.0
PY	31.7 ± 28.8	31.6 ± 28.9	35.1 ± 25.8	0.683
Discharge (days)				
In-hospital stay	8.5 ± 4.7	8.48 ± 4.7	9.25 ± 5.1	0.563

Abbreviations: BMI: Body Mass Index; COPD: Chronic Obstructive Pulmonary Disease; CHD: Coronary Heart Disease; PY: Pack-Year.

**Table 2:** Oncological baseline characteristics after PSM.

	Total	ADC	LCNEC	*p*-Value
UICC stage (%)				
I	43.8	50.0	37.5	0.722
II	25.0	25.0	25.0	1.0
III	31.2	25.0	37.5	0.704
Blood-vessel invasion (%)				
V1	28.1	25.0	31.3	1.0
Lymphatic-vessel invasion (%)				
L1	34.4	31.3	37.5	1.0
LN-involvement (%)				
pN0	50.0	56.2	43.8	
pN1	46.9	43.8	50.0	
pN2	3.1	0	6.2	0.358

Abbreviations: LN: Lymph Node.

**Table 3:** Mean and long-term survival rates of ADC vs. LCNEC before and after PSM.

Survival Before PSM	ADC	LCNEC	*p*-Value
3-year survival (%)	59.9	27.3	0.033
Mean-survival (Months)	62.5 ± 2.3	36.4 ± 7.5	0.011
Survival after PSM			
3-year survival (%)	92.9	27.3	0.002
Mean-survival (Months)	80.7 ± 8.1	36.4 ± 7.5	0.001

**Table 4:** Baseline characteristics of LCNEC CK7+/− patients.

	Total	CK7+ (*n* = 10)	CK7− (*n* = 6)	*p*-Value
Age	66.6 ± 10.9	69.1 ± 11.0	62.5 ± 10.5	0.258
UICC stage (%)				
I	37.5	40.0	33.3	1.0
II	25.0	20.0	33.3	0.604
III	37.5	40.0	33.3	1.0
Blood-vessel invasion (%)				
V1	37.5	40.0	33.0	1.0
Lymphatic-vessel invasion (%)				
L1	43.8	40.0	50.0	1.0
LN-involvement (%)				
pN0	43.8	40.0	50.0	
pN1	37.5	40.0	33.3	
pN2	18.8	20.0	16.7	0.751
Synaptophysin (%)				
positive	56.3	50.0	66.6	1.0
Chromogranin A (%)				
positive	31.3	10.0	66.6	0.08
TTF1 (%)				
expression	50.0	55.6	40.0	1.0

Abbreviations: TTF1: Thyroid transcription factor 1.

**Table 5:** Survival and Cox Regression Analysis of CK7+ vs. CK7− LCNEC patients.

Total (*n* = 16)	CK7+ (*n* = 10)	CK7− (*n* = 6)	*p*-Value
3-year survival (%)	12.5	75.0	0.067
Mean-survival (Months)	19.23 ± 4.8	57.01 ± 8.5	0.032
	*p*-value	C.I.	HR
Cox Regression Analysis	0.014	1.0–77.9	9.02

Abbreviations: C.I.: Confidence Interval; HR: Hazard Ratio.

## Data Availability

The data underlying this article will be shared upon reasonable request to the corresponding author.

## Abbreviations

LCNEC: Large-Cell Neuroendocrine Carcinoma; SCLC: Small-Cell Lung Cancer; NSCLC: Non-Small-Cell Lung Cancer; ADC: Adenocarcinoma; NCCN: National Comprehensive Cancer Network; Hr-CT: High-Resolution computed tomography; PET-CT: positron emission tomography computed tomography; DLCO: diffusing capacity of carbon monoxide; FEV1: forced expiratory volume in one second; MDT: Multidisciplinary tumor conference; CK7: Cytokeratin 7; NET: Neuroendocrine tumors; NEN: Neuroendocrine Neoplasms; NEC: Neuroendocrine Carcinoma.
